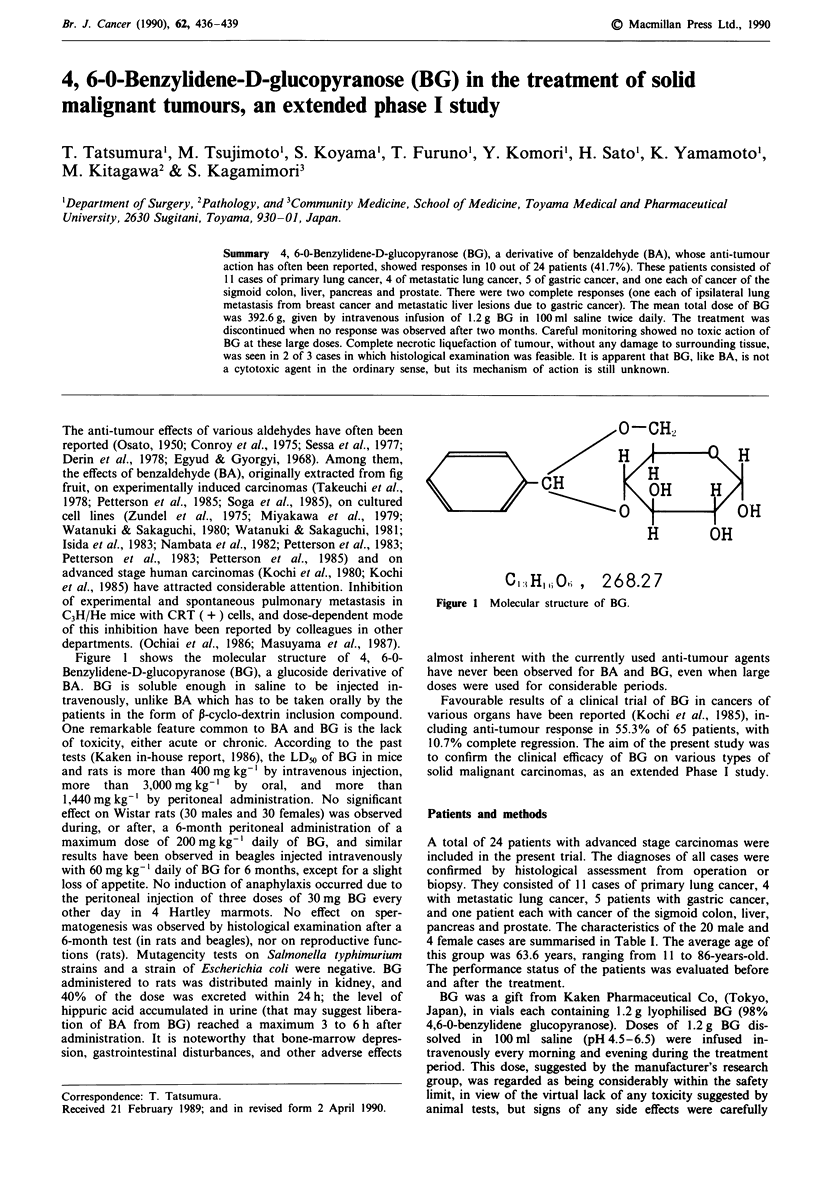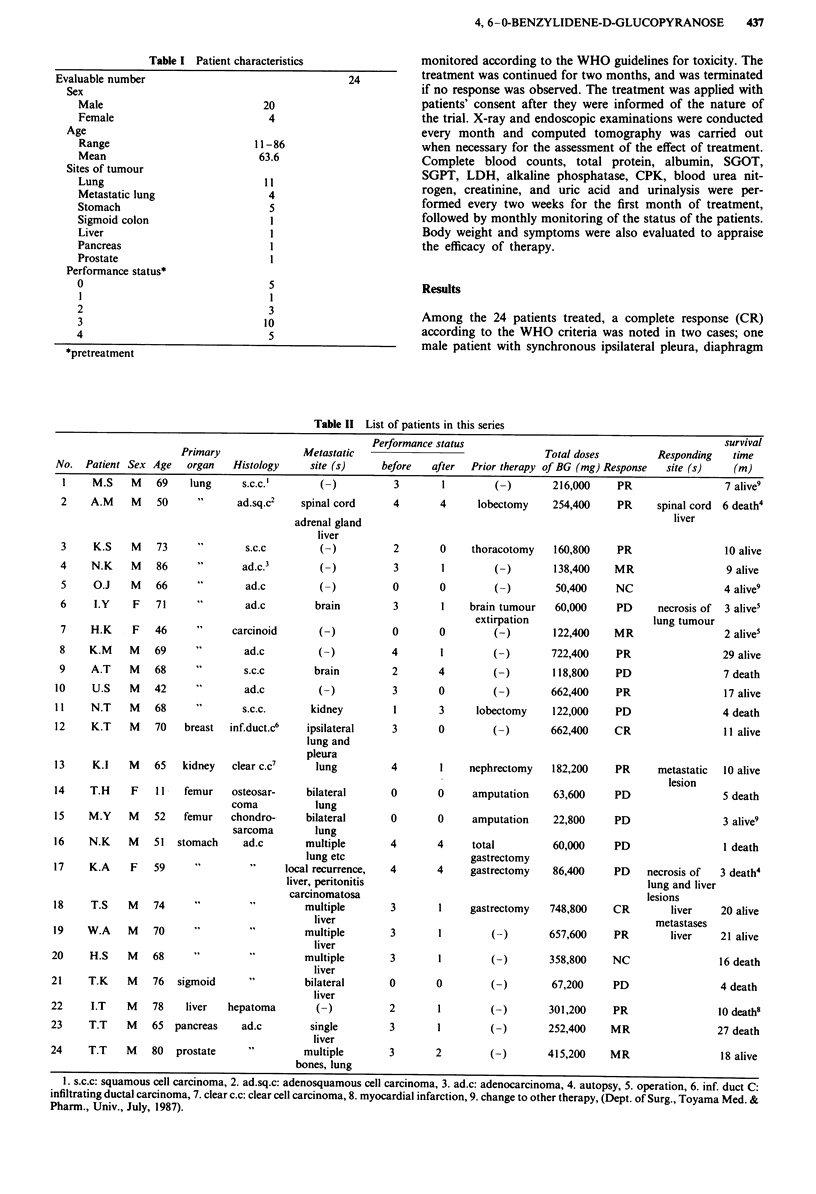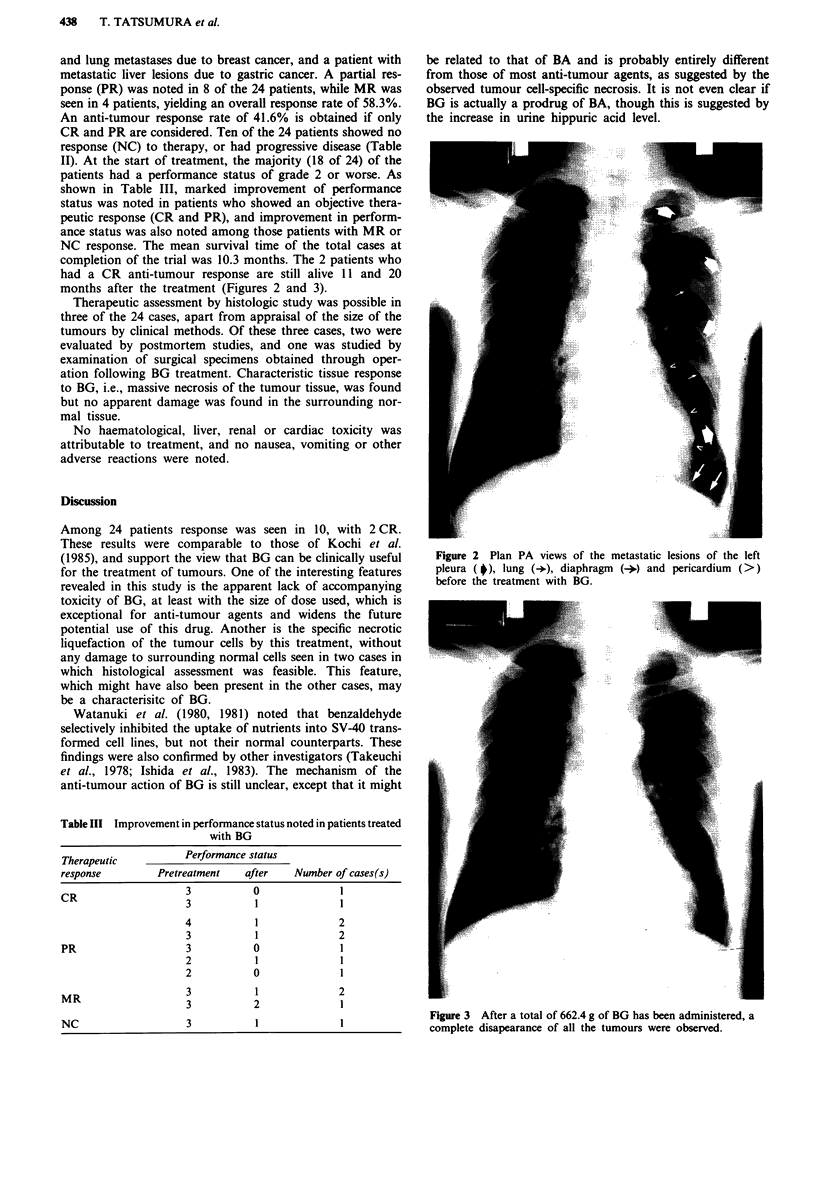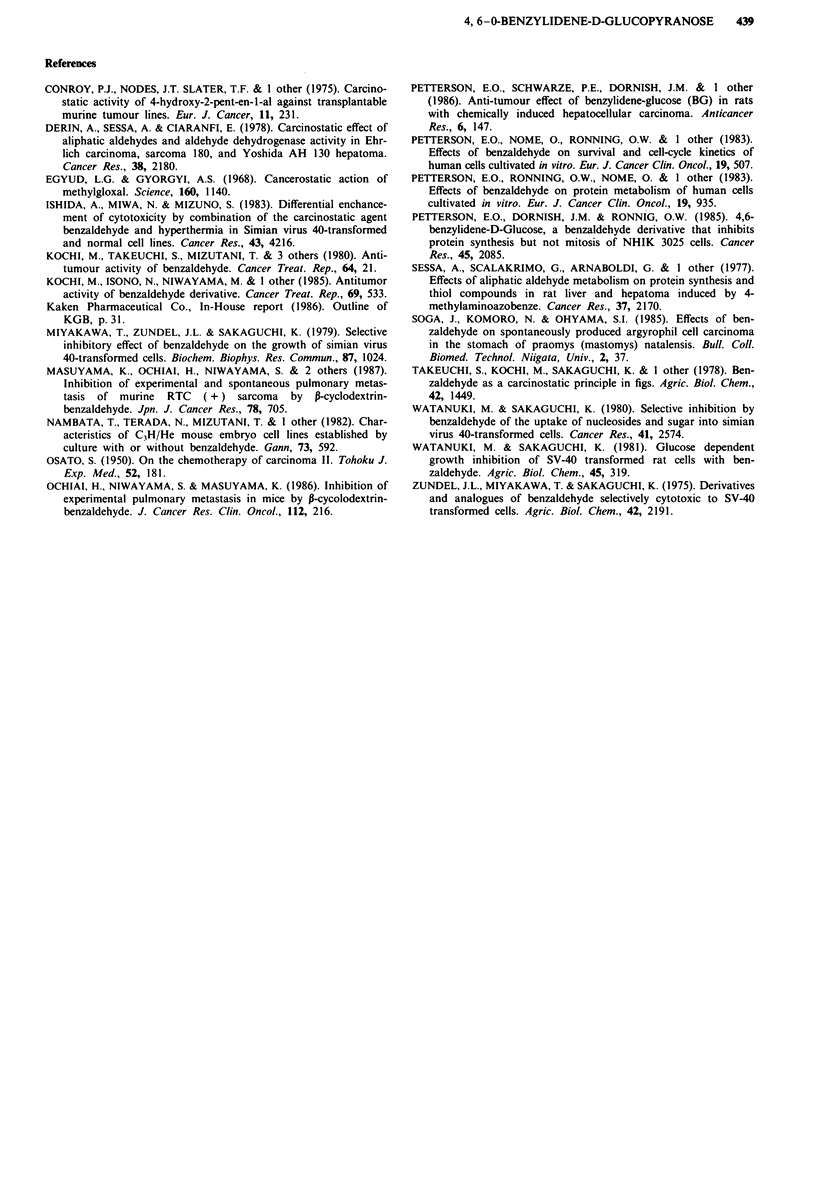# 4, 6-0-benzylidene-D-glucopyranose (BG) in the treatment of solid malignant tumours, an extended phase I study.

**DOI:** 10.1038/bjc.1990.313

**Published:** 1990-09

**Authors:** T. Tatsumura, M. Tsujimoto, S. Koyama, T. Furuno, Y. Komori, H. Sato, K. Yamamoto, M. Kitagawa, S. Kagamimori

**Affiliations:** Department of Surgery, School of Medicine, Toyama Medical and Pharmaceutical University, Japan.

## Abstract

**Images:**


Br. J. Cancer (1990), 62, 436-439 © Macmillan Press Ltd., 1990

### 4, 6-0-Benzylidene-D-glucopyranose (BG) in the treatment of solid malignant tumours, an extended phase I study

T. Tatsumura[1], M. Tsujimoto[1], S. Koyama[1], T. Furuno[1], Y. Komori[1], H. Sato[1], K. Yamamoto[1], M. Kitagawa[2] & S. Kagamimori[3]

[1]*Department of Surgery,* [2]*Pathology, and* [3]*Community Medicine, School of Medicine, Toyama Medical and Pharmaceutical University, 2630 Sugitani, Toyama, 930-01, Japan.*

**Summary** 4, 6-0-Benzylidene-D-glucopyranose (BG), a derivative of benzaldehyde (BA), whose anti-tumour action has often been reported, showed responses in 10 out of 24 patients (41.7%). These patients consisted of 11 cases of primary lung cancer, 4 of metastatic lung cancer, 5 of gastric cancer, and one each of cancer of the sigmoid colon, liver, pancreas and prostate. There were two complete responses (one each of ipsilateral lung metastasis from breast cancer and metastatic liver lesions due to gastric cancer). The mean total dose of BG was 392.6 g, given by intravenous infusion of 1.2 g BG in 100 ml saline twice daily. The treatment was discontinued when no response was observed after two months. Careful monitoring showed no toxic action of BG at these large doses. Complete necrotic liquefaction of tumour, without any damage to surrounding tissue, was seen in 2 of 3 cases in which histological examination was feasible. It is apparent that BG, like BA, is not a cytotoxic agent in the ordinary sense, but its mechanism of action is still unknown.

The anti-tumour effects of various aldehydes have often been reported (Osato, 1950; Conroy *et al.*, 1975; Sessa *et al.*, 1977; Derin *et al.*, 1978; Egyud & Gyorgyi, 1968). Among them, the effects of benzaldehyde (BA), originally extracted from fig fruit, on experimentally induced carcinomas (Takeuchi *et al.*, 1978; Petterson *et al.*, 1985; Soga *et al.*, 1985), on cultured cell lines (Zundel *et al.*, 1975; Miyakawa *et al.*, 1979; Watanuki & Sakaguchi, 1980; Watanuki & Sakaguchi, 1981; Isida *et al.*, 1983; Nambata *et al.*, 1982; Petterson *et al.*, 1983; Petterson *et al.*, 1983; Petterson *et al.*, 1985) and on advanced stage human carcinomas (Kochi *et al.*, 1980; Kochi *et al.*, 1985) have attracted considerable attention. Inhibition of experimental and spontaneous pulmonary metastasis in $C_3H/He$ mice with CRT ( + ) cells, and dose-dependent mode of this inhibition have been reported by colleagues in other departments. (Ochiai *et al.*, 1986; Masuyama *et al.*, 1987).

Figure 1 shows the molecular structure of 4, 6-0-Benzylidene-D-glucopyranose (BG), a glucoside derivative of BA. BG is soluble enough in saline to be injected intravenously, unlike BA which has to be taken orally by the patients in the form of β-cyclo-dextrin inclusion compound. One remarkable feature common to BA and BG is the lack of toxicity, either acute or chronic. According to the past tests (Kaken in-house report, 1986), the $LD_{50}$ of BG in mice and rats is more than 400 mg kg$^{-1}$ by intravenous injection, more than 3,000 mg kg$^{-1}$ by oral, and more than 1,440 mg kg$^{-1}$ by peritoneal administration. No significant effect on Wistar rats (30 males and 30 females) was observed during, or after, a 6-month peritoneal administration of a maximum dose of 200 mg kg$^{-1}$ daily of BG, and similar results have been observed in beagles injected intravenously with 60 mg kg$^{-1}$ daily of BG for 6 months, except for a slight loss of appetite. No induction of anaphylaxis occurred due to the peritoneal injection of three doses of 30 mg BG every other day in 4 Hartley marmots. No effect on spermatogenesis was observed by histological examination after a 6-month test (in rats and beagles), nor on reproductive functions (rats). Mutagencity tests on *Salmonella typhimurium* strains and a strain of *Escherichia coli* were negative. BG administered to rats was distributed mainly in kidney, and 40% of the dose was excreted within 24 h; the level of hippuric acid accumulated in urine (that may suggest liberation of BA from BG) reached a maximum 3 to 6 h after administration. It is noteworthy that bone-marrow depression, gastrointestinal disturbances, and other adverse effects

$C_{13}H_{16}O_6$ , 268.27

**Figure 1** Molecular structure of BG.

almost inherent with the currently used anti-tumour agents have never been observed for BA and BG, even when large doses were used for considerable periods.

Favourable results of a clinical trial of BG in cancers of various organs have been reported (Kochi *et al.*, 1985), including anti-tumour response in 55.3% of 65 patients, with 10.7% complete regression. The aim of the present study was to confirm the clinical efficacy of BG on various types of solid malignant carcinomas, as an extended Phase I study.

### Patients and methods

A total of 24 patients with advanced stage carcinomas were included in the present trial. The diagnoses of all cases were confirmed by histological assessment from operation or biopsy. They consisted of 11 cases of primary lung cancer, 4 with metastatic lung cancer, 5 patients with gastric cancer, and one patient each with cancer of the sigmoid colon, liver, pancreas and prostate. The characteristics of the 20 male and 4 female cases are summarised in Table I. The average age of this group was 63.6 years, ranging from 11 to 86-years-old. The performance status of the patients was evaluated before and after the treatment.

BG was a gift from Kaken Pharmaceutical Co, (Tokyo, Japan), in vials each containing 1.2 g lyophilised BG (98% 4,6-0-benzylidene glucopyranose). Doses of 1.2 g BG dissolved in 100 ml saline (pH 4.5-6.5) were infused intravenously every morning and evening during the treatment period. This dose, suggested by the manufacturer's research group, was regarded as being considerably within the safety limit, in view of the virtual lack of any toxicity suggested by animal tests, but signs of any side effects were carefully

Correspondence: T. Tatsumura.
Received 21 February 1989; and in revised form 2 April 1990.

**Table I** Patient characteristics

| | |
|---|---|
| Evaluable number | 24 |
| Sex | |
| Male | 20 |
| Female | 4 |
| Age | |
| Range | 11-86 |
| Mean | 63.6 |
| Sites of tumour | |
| Lung | 11 |
| Metastatic lung | 4 |
| Stomach | 5 |
| Sigmoid colon | 1 |
| Liver | 1 |
| Pancreas | 1 |
| Prostate | 1 |
| Performance status* | |
| 0 | 5 |
| 1 | 1 |
| 2 | 3 |
| 3 | 10 |
| 4 | 5 |

*pretreatment monitored according to the WHO guidelines for toxicity. The treatment was continued for two months, and was terminated if no response was observed. The treatment was applied with patients' consent after they were informed of the nature of the trial. X-ray and endoscopic examinations were conducted every month and computed tomography was carried out when necessary for the assessment of the effect of treatment. Complete blood counts, total protein, albumin, SGOT, SGPT, LDH, alkaline phosphatase, CPK, blood urea nitrogen, creatinine, and uric acid and urinalysis were performed every two weeks for the first month of treatment, followed by monthly monitoring of the status of the patients. Body weight and symptoms were also evaluated to appraise the efficacy of therapy.

### Results

Among the 24 patients treated, a complete response (CR) according to the WHO criteria was noted in two cases; one male patient with synchronous ipsilateral pleura, diaphragm

**Table II** List of patients in this series

| No. | Patient | Sex | Age | Primary organ | Histology | Metastatic site (s) | Performance status before | Performance status after | Prior therapy | Total doses of BG (mg) | Response | Responding site (s) | survival time (m) |
|---|---|---|---|---|---|---|---|---|---|---|---|---|---|
| 1 | M.S | M | 69 | lung | s.c.c.[1] | (-) | 3 | 1 | (-) | 216,000 | PR | | 7 alive[9] |
| 2 | A.M | M | 50 | " | ad.sq.c[2] | spinal cord adrenal gland liver | 4 | 4 | lobectomy | 254,400 | PR | spinal cord liver | 6 death[4] |
| 3 | K.S | M | 73 | " | s.c.c | (-) | 2 | 0 | thoracotomy | 160,800 | PR | | 10 alive |
| 4 | N.K | M | 86 | " | ad.c.[3] | (-) | 3 | 1 | (-) | 138,400 | MR | | 9 alive |
| 5 | O.J | M | 66 | " | ad.c | (-) | 0 | 0 | (-) | 50,400 | NC | | 4 alive[9] |
| 6 | I.Y | F | 71 | " | ad.c | brain | 3 | 1 | brain tumour extirpation | 60,000 | PD | necrosis of lung tumour | 3 alive[5] |
| 7 | H.K | F | 46 | " | carcinoid | (-) | 0 | 0 | (-) | 122,400 | MR | | 2 alive[5] |
| 8 | K.M | M | 69 | " | ad.c | (-) | 4 | 1 | (-) | 722,400 | PR | | 29 alive |
| 9 | A.T | M | 68 | " | s.c.c | brain | 2 | 4 | (-) | 118,800 | PD | | 7 death |
| 10 | U.S | M | 42 | " | ad.c | (-) | 3 | 0 | (-) | 662,400 | PR | | 17 alive |
| 11 | N.T | M | 68 | " | s.c.c. | kidney | 1 | 3 | lobectomy | 122,000 | PD | | 4 death |
| 12 | K.T | M | 70 | breast | inf.duct.c[6] | ipsilateral lung and pleura | 3 | 0 | (-) | 662,400 | CR | | 11 alive |
| 13 | K.I | M | 65 | kidney | clear c.c[7] | lung | 4 | 1 | nephrectomy | 182,200 | PR | metastatic lesion | 10 alive |
| 14 | T.H | F | 11 | femur | osteosarcoma | bilateral lung | 0 | 0 | amputation | 63,600 | PD | | 5 death |
| 15 | M.Y | M | 52 | femur | chondrosarcoma | bilateral lung | 0 | 0 | amputation | 22,800 | PD | | 3 alive[9] |
| 16 | N.K | M | 51 | stomach | ad.c | multiple lung etc | 4 | 4 | total gastrectomy | 60,000 | PD | | 1 death |
| 17 | K.A | F | 59 | " | " | local recurrence, liver, peritonitis carcinomatosa | 4 | 4 | gastrectomy | 86,400 | PD | necrosis of lung and liver lesions | 3 death[4] |
| 18 | T.S | M | 74 | " | " | multiple liver | 3 | 1 | gastrectomy | 748,800 | CR | liver metastases | 20 alive |
| 19 | W.A | M | 70 | " | " | multiple liver | 3 | 1 | (-) | 657,600 | PR | liver | 21 alive |
| 20 | H.S | M | 68 | " | " | multiple liver | 3 | 1 | (-) | 358,800 | NC | | 16 death |
| 21 | T.K | M | 76 | sigmoid | " | bilateral liver | 0 | 0 | (-) | 67,200 | PD | | 4 death |
| 22 | I.T | M | 78 | liver | hepatoma | (-) | 2 | 1 | (-) | 301,200 | PR | | 10 death[8] |
| 23 | T.T | M | 65 | pancreas | ad.c | single liver | 3 | 1 | (-) | 252,400 | MR | | 27 death |
| 24 | T.T | M | 80 | prostate | " | multiple bones, lung | 3 | 2 | (-) | 415,200 | MR | | 18 alive |

1. s.c.c: squamous cell carcinoma, 2. ad.sq.c: adenosquamous cell carcinoma, 3. ad.c: adenocarcinoma, 4. autopsy, 5. operation, 6. inf. duct C: infiltrating ductal carcinoma, 7. clear c.c: clear cell carcinoma, 8. myocardial infarction, 9. change to other therapy, (Dept. of Surg., Toyama Med. & Pharm., Univ., July, 1987).

and lung metastases due to breast cancer, and a patient with metastatic liver lesions due to gastric cancer. A partial response (PR) was noted in 8 of the 24 patients, while MR was seen in 4 patients, yielding an overall response rate of 58.3%. An anti-tumour response rate of 41.6% is obtained if only CR and PR are considered. Ten of the 24 patients showed no response (NC) to therapy, or had progressive disease (Table II). At the start of treatment, the majority (18 of 24) of the patients had a performance status of grade 2 or worse. As shown in Table III, marked improvement of performance status was noted in patients who showed an objective therapeutic response (CR and PR), and improvement in performance status was also noted among those patients with MR or NC response. The mean survival time of the total cases at completion of the trial was 10.3 months. The 2 patients who had a CR anti-tumour response are still alive 11 and 20 months after the treatment (Figures 2 and 3).

Therapeutic assessment by histologic study was possible in three of the 24 cases, apart from appraisal of the size of the tumours by clinical methods. Of these three cases, two were evaluated by postmortem studies, and one was studied by examination of surgical specimens obtained through operation following BG treatment. Characteristic tissue response to BG, i.e., massive necrosis of the tumour tissue, was found but no apparent damage was found in the surrounding normal tissue.

No haematological, liver, renal or cardiac toxicity was attributable to treatment, and no nausea, vomiting or other adverse reactions were noted.

### Discussion

Among 24 patients response was seen in 10, with 2 CR. These results were comparable to those of Kochi *et al.* (1985), and support the view that BG can be clinically useful for the treatment of tumours. One of the interesting features revealed in this study is the apparent lack of accompanying toxicity of BG, at least with the size of dose used, which is exceptional for anti-tumour agents and widens the future potential use of this drug. Another is the specific necrotic liquefaction of the tumour cells by this treatment, without any damage to surrounding normal cells seen in two cases in which histological assessment was feasible. This feature, which might have also been present in the other cases, may be a characterisitc of BG.

Watanuki *et al.* (1980, 1981) noted that benzaldehyde selectively inhibited the uptake of nutrients into SV-40 transformed cell lines, but not their normal counterparts. These findings were also confirmed by other investigators (Takeuchi *et al.*, 1978; Ishida *et al.*, 1983). The mechanism of the anti-tumour action of BG is still unclear, except that it might be related to that of BA and is probably entirely different from those of most anti-tumour agents, as suggested by the observed tumour cell-specific necrosis. It is not even clear if BG is actually a prodrug of BA, though this is suggested by the increase in urine hippuric acid level.

**Table III** Improvement in performance status noted in patients treated with BG

| *Therapeutic response* | *Performance status* | | |
|---|---|---|---|
| | *Pretreatment* | *after* | *Number of cases(s)* |
| CR | 3 | 0 | 1 |
| | 3 | 1 | 1 |
| PR | 4 | 1 | 2 |
| | 3 | 1 | 2 |
| | 3 | 0 | 1 |
| | 2 | 1 | 1 |
| | 2 | 0 | 1 |
| MR | 3 | 1 | 2 |
| | 3 | 2 | 1 |
| NC | 3 | 1 | 1 |

**Figure 2** Plan PA views of the metastatic lesions of the left pleura (⧫), lung (→), diaphragm (→) and pericardium (>) before the treatment with BG.

**Figure 3** After a total of 662.4 g of BG has been administered, a complete disapearance of all the tumours were observed.

**References**

CONROY, P.J., NODES, J.T. SLATER, T.F. & 1 other (1975). Carcinostatic activity of 4-hydroxy-2-pent-en-1-al against transplantable murine tumour lines. *Eur. J. Cancer*, **11**, 231.

DERIN, A., SESSA, A. & CIARANFI, E. (1978). Carcinostatic effect of aliphatic aldehydes and aldehyde dehydrogenase activity in Ehrlich carcinoma, sarcoma 180, and Yoshida AH 130 hepatoma. *Cancer Res.*, **38**, 2180.

EGYUD, L.G. & GYORGYI, A.S. (1968). Cancerostatic action of methylgloxal. *Science*, **160**, 1140.

ISHIDA, A., MIWA, N. & MIZUNO, S. (1983). Differential enchancement of cytotoxicity by combination of the carcinostatic agent benzaldehyde and hyperthermia in Simian virus 40-transformed and normal cell lines. *Cancer Res.*, **43**, 4216.

KOCHI, M., TAKEUCHI, S., MIZUTANI, T. & 3 others (1980). Antitumour activity of benzaldehyde. *Cancer Treat. Rep.*, **64**, 21.

KOCHI, M., ISONO, N., NIWAYAMA, M. & 1 other (1985). Antitumor activity of benzaldehyde derivative. *Cancer Treat. Rep.*, **69**, 533.

Kaken Pharmaceutical Co., In-House report (1986). Outline of KGB, p. 31.

MIYAKAWA, T., ZUNDEL, J.L. & SAKAGUCHI, K. (1979). Selective inhibitory effect of benzaldehyde on the growth of simian virus 40-transformed cells. *Biochem. Biophys. Res. Commun.*, **87**, 1024.

MASUYAMA, K., OCHIAI, H., NIWAYAMA, S. & 2 others (1987). Inhibition of experimental and spontaneous pulmonary metastasis of murine RTC (+) sarcoma by β-cyclodextrin-benzaldehyde. *Jpn. J. Cancer Res.*, **78**, 705.

NAMBATA, T., TERADA, N., MIZUTANI, T. & 1 other (1982). Characteristics of $C_3H$/He mouse embryo cell lines established by culture with or without benzaldehyde. *Gann*, **73**, 592.

OSATO, S. (1950). On the chemotherapy of carcinoma II. *Tohoku J. Exp. Med.*, **52**, 181.

OCHIAI, H., NIWAYAMA, S. & MASUYAMA, K. (1986). Inhibition of experimental pulmonary metastasis in mice by β-cycolodextrin-benzaldehyde. *J. Cancer Res. Clin. Oncol.*, **112**, 216.

PETTERSON, E.O., SCHWARZE, P.E., DORNISH, J.M. & 1 other (1986). Anti-tumour effect of benzylidene-glucose (BG) in rats with chemically induced hepatocellular carcinoma. *Anticancer Res.*, **6**, 147.

PETTERSON, E.O., NOME, O., RONNING, O.W. & 1 other (1983). Effects of benzaldehyde on survival and cell-cycle kinetics of human cells cultivated *in vitro*. *Eur. J. Cancer Clin. Oncol.*, **19**, 507.

PETTERSON, E.O., RONNING, O.W., NOME, O. & 1 other (1983). Effects of benzaldehyde on protein metabolism of human cells cultivated *in vitro*. *Eur. J. Cancer Clin. Oncol.*, **19**, 935.

PETTERSON, E.O., DORNISH, J.M. & RONNIG, O.W. (1985). 4,6-benzylidene-D-Glucose, a benzaldehyde derivative that inhibits protein synthesis but not mitosis of NHIK 3025 cells. *Cancer Res.*, **45**, 2085.

SESSA, A., SCALAKRIMO, G., ARNABOLDI, G. & 1 other (1977). Effects of aliphatic aldehyde metabolism on protein synthesis and thiol compounds in rat liver and hepatoma induced by 4-methylaminoazobenze. *Cancer Res.*, **37**, 2170.

SOGA, J., KOMORO, N. & OHYAMA, S.I. (1985). Effects of benzaldehyde on spontaneously produced argyrophil cell carcinoma in the stomach of praomys (mastomys) natalensis. *Bull. Coll. Biomed. Technol. Niigata, Univ.*, **2**, 37.

TAKEUCHI, S., KOCHI, M., SAKAGUCHI, K. & 1 other (1978). Benzaldehyde as a carcinostatic principle in figs. *Agric. Biol. Chem.*, **42**, 1449.

WATANUKI, M. & SAKAGUCHI, K. (1980). Selective inhibition by benzaldehyde of the uptake of nucleosides and sugar into simian virus 40-transformed cells. *Cancer Res.*, **41**, 2574.

WATANUKI, M. & SAKAGUCHI, K. (1981). Glucose dependent growth inhibition of SV-40 transformed rat cells with benzaldehyde. *Agric. Biol. Chem.*, **45**, 319.

ZUNDEL, J.L., MIYAKAWA, T. & SAKAGUCHI, K. (1975). Derivatives and analogues of benzaldehyde selectively cytotoxic to SV-40 transformed cells. *Agric. Biol. Chem.*, **42**, 2191.